# Protocol for a systematic review of autologous fat grafting for wound healing

**DOI:** 10.1186/s13643-018-0769-7

**Published:** 2018-07-18

**Authors:** Joshua Luck, Oliver J. Smith, Dean Malik, Afshin Mosahebi

**Affiliations:** 1 UCL Division of Surgery and Interventional Science, Royal Free Hospital, 9th Floor, Pond Street, London, NW3 2QG UK; 2 Department of Plastic and Reconstructive Surgery, Royal Free Hospital, London, UK

**Keywords:** Adipose-derived stem cells, Autologous fat, Cell-assisted lipotransfer, Fat grafting, Lipotransfer, Stromal vascular fraction, Wound healing, Ulcer, Systematic review

## Abstract

**Background:**

Autologous fat grafting is an emerging therapeutic option for cutaneous wounds. The regenerative potential of autologous fat relates to the presence of adipose-derived stem cells (ADSCs) within the stromal vascular fraction (SVF). ADSCs are capable of differentiating into fibroblasts and keratinocytes, as well as secreting soluble mediators with angiogenic and anti-inflammatory properties. However, to date, there has been no comprehensive assessment of the wound healing literature in humans. This systematic review aims to critically evaluate the efficacy and safety of autologous fat grafting in acute and chronic cutaneous wounds with an appraisal of the quality of evidence available.

**Methods:**

Following PRISMA guidelines, MEDLINE, Embase and Cochrane Library databases will be searched from inception to December 2017. All primary clinical studies in which wounds are treated with lipotransfer, cell-assisted lipotransfer (CAL), SVF products or isolated ADSCs will be eligible for inclusion. Study screening and data extraction will be conducted by two authors in duplicate. Our primary outcome measure will be the proportion of completely healed wounds at 12 weeks. Secondary outcome measures will include the proportion of partially healed wounds at 12 weeks; the mean wound surface area reduction at 12 weeks; the mean time to wound healing; and adverse event rates. The quality of evidence for each summary outcome measure will be assessed using the GRADE approach.

**Discussion:**

In light of the growing popularity of autologous fat grafting for wound healing, a systematic appraisal of the available evidence is timely. If autologous fat grafting is associated with a positive treatment effect, we will compare these outcomes to those achieved using alternative wound management strategies. This review also aims to determine if one or more autologous fat grafting techniques are superior and whether this varies according to patient- and wound-specific factors. We anticipate that these results will guide future research and inform clinical practice.

**Systematic review registration:**

PROSPERO CRD42017081499

## Background

There is growing interest in the regenerative potential of autologous fat grafting (AFG). Adipose-derived stem cells (ADSCs) contained within the stromal vascular fraction (SVF) of lipoaspirate samples promote revascularisation, activate local stem cell niches and modify immune responses via the paracrine secretion of numerous bioactive molecules [1, 2]. They are also able to differentiate into various terminal phenotypes that contribute to wound healing, including fibroblasts and keratinocytes [3]. Unlike many other mesenchymal stem cells (MSCs), including bone marrow MSCs, ADSCs may be harvested with minimal donor site morbidity and used without ex vivo culturing and expansion [4]. Autologous ADSCs do not provoke a foreign body response, and they are found in the greatest concentration of any MSCs in the body (up to 5000 cells may be isolated per gram of adipose tissue [3, 5]).

The most common method of AFG is based on the Coleman technique [6]. Here, donor site lipoaspirate samples are harvested and processed prior to injection into a recipient site. The standard technique is referred to as lipotransfer, although several variations exist. For example, the supplementation of harvested lipoaspirate with either the mixed cellular SVF milieu (containing ADSCs, preadipocytes, endothelial cells, haematopoetic-lineage cells and fibroblasts [7]) or isolated ADSCs may enhance the regenerative potential of AFG [8]. This technique is called cell-assisted lipotransfer (CAL). Alternatively, the SVF cell pellet or a pure ADSC population may be injected without lipoaspirate reconstitution [9–11]. There is no consensus on the terminology for SVF- and ADSC-only therapy; for the purposes of this review, we refer to these approaches as SVF or ADSC monotherapy.

Although the early evidence for AFG for wound healing is promising, much of the literature is based on animal studies and there has been no comprehensive evaluation of its efficacy and safety in humans. Similarly, there have been no attempts made to systematically appraise the quality of the evidence across the literature, despite the growing popularity of AFG in the clinical setting [12]. Finally, there has been no comparative assessment of the different AFG techniques for wound healing and it is unclear whether one or more approaches are superior.

This systematic review aims to critically evaluate the effectiveness and safety profile of AFG for acute and chronic cutaneous wounds of any aetiology with an assessment of the quality of the evidence available. It also seeks to determine if any one AFG technique leads to better outcomes and whether this differs according to either wound or patient characteristics. A secondary objective will be to compare AFG results to those obtained using alternative wound management options.

## Methods

This protocol has been prospectively registered on the PROSPERO database (CRD42017081499) and will be reported according to the Preferred Reporting Items for Systematic Review and Meta-Analyses Protocols (PRISMA-P) statement [13]. The final review will be conducted and reported with reference to the PRISMA statement [14]. In the event of no randomised controlled trial (RCT) being included, additional consideration will be given to the Meta-Analysis of Observational Studies in Epidemiology (MOOSE) guidelines [15].

### Search strategy

We will search MEDLINE, Embase and Cochrane Library databases from inception to December 2017. Both free-text terms and MeSH headings will be combined with Boolean operators (see Table 1). A stepwise free-text term serial search example is provided in the Appendix.

**Table 1:** Electronic database search strategy

Free-text terms		MeSH headings
fat graft*	wound heal*	Adipose tissue
fat transf*	wound management	Lipectomy
fat transplant*	wound treat*	Skin ulcer
fat inject*	ulcer heal*	Transplantation, autologous
adipose graft*	ulcer management*	Wound healing
adipose stem cell*	ulcer treat*	
adipose derived stem cell*		
adipose transplant*		
ASC*		
ADSC*		
lipofill*		
lipotransf*		
lipomodell*		

After removing duplicate citations, titles and abstracts will be screened by two authors (DM and JL) in parallel. The remaining articles will be read in full to shortlist articles for inclusion. The reference lists of all included studies will be routinely checked to ensure that no relevant studies have been missed. English language publications from any country of origin will be eligible for inclusion. Citations will be managed using Mendeley (Elsevier, Amsterdam, Netherlands) and Excel (Microsoft, Redmond, Washington, USA).

### Study selection criteria

All primary clinical studies using autologous fat therapy for treating cutaneous wounds of any age will be eligible for inclusion. For the purposes of this study, we define a wound as any loss in epithelial continuity (with or without involvement of underlying connective tissue). Healed scars will not be considered as wounds.

#### Participants

Randomised controlled and observational studies conducted in any clinical setting involving three or more adult participants (aged between 18 and 90 years old) will be eligible for inclusion. No restrictions will be applied to the following: wound aetiology, wound size, wound chronicity or number of wounds per patient. Case reports, letters, editorials, conference abstracts and literature reviews will be excluded.

#### Intervention

All methods in which autologous fat or its derivatives are applied to a wound will be deemed eligible for inclusion. AFG interventions will include conventional fat grafting (lipotransfer), CAL and isolated ADSC or SVF monotherapy. No restrictions will be applied to the following: fat harvesting technique, fat processing methodology, in vitro cell expansion, number of interventions or wound bed preparation. All adjunct therapies will be included, with the exception of platelet-rich plasma (PRP) as this has already been reviewed by our research group (*in press*). Studies in which PRP and autologous fat are co-administered will be excluded from this review.

#### Comparator

All experimental studies evaluating AFG either in isolation, in conjunction or in comparison to any other wound management option will be eligible for inclusion. We intend to compare AFG outcomes with alternative wound management options. However, to avoid misleading cross-comparative evaluation of the literature, we will only extract data from comparator interventions or controls when these have been conducted in parallel with and directly compared to an AFG treatment arm.

#### Outcome

Studies reporting on any clinically relevant outcome at any length of follow-up will be eligible for inclusion. Ongoing and unpublished studies will be excluded.

### Data extraction

Data collection and analysis will be completed as per the Cochrane Handbook of Systematic Reviews of Interventions [16]. All data will be extracted onto a pre-designed electronic form in duplicate by two authors (JL and DM) to ensure accuracy. Any disagreements will be resolved by discussion and consensus with referral to a third review team member (OJS) as necessary.

Data items will relate to the following:

1. Study design and patient demographics
2. Pre-intervention wound characteristics
3. Autologous fat therapy methodology
4. Post-intervention wound healing outcomes

Where studies provide information on multiple different interventions, data will be extracted for those related to the current research objective only. Where appropriate, authors will be contacted to provide missing information.

The unit of analysis will be individual wounds. For example, if AFG is used to treat multiple wounds on the same patient, data will be extracted per wound.

### Outcome measures

Our primary outcome will be the proportion of completely healed wounds at 12 weeks.

Secondary outcome measures will include the proportion of partially healed wounds at 12 weeks (defined as a 1–99% reduction in wound surface area); the mean wound surface area reduction at 12 weeks (as a percentage change from baseline); the time to complete wound healing (defined as complete re-epithelialisation); and adverse event rates (related to either donor or recipient sites).

If three or more included studies report on either economic or health-related quality of life outcomes, we will extract these data and comment on its implications in the qualitative synthesis.

### Subgroup analyses

Subgroup analyses will be performed to further interrogate primary and/or secondary outcomes based on wound aetiology (e.g. diabetic wounds, traumatic wounds, venous wounds, arterial wounds, neuropathic wounds, burn wounds) and chronicity (with acute defined as < 3 months and chronic > 3 months duration) providing sufficient data are available.

To ascertain if any single method of AFG is superior, we will present data according to the intervention type used (i.e. lipotransfer, CAL, SVF- or ADSC-only groups).

### Risk of bias assessment

We anticipate that all included studies will use an observational study design. Risk of bias for each study will therefore be assessed using the ROBINS-I tool [17]. However, if any randomised controlled trials (RCTs) are included, these will also be further assessed on a per study basis using the Cochrane Collaboration Risk of Bias Assessment Tool [18].

Two authors (JL and DM) will independently appraise the quality of evidence across all included studies for each outcome of interest using the Grading of Recommendations, Assessment, Development and Evaluation (GRADE) approach [19]. Any discrepancies will be resolved by discussion and consensus or referral to a third team member (OJS or AM) as necessary.

### Data analysis and synthesis

Primary and secondary outcome measures will be evaluated using simple descriptive statistics. Where appropriate, we will provide pooled estimates with corresponding measures of dispersion.

We will only perform a meta-analysis if a sufficient number of studies (minimum ≥ 3) with consistent characteristics are included. In the first instance, we will combine all AFG techniques into a single intervention group. If ≥ 3 comparable studies are available for one or more intervention subtypes (namely lipotransfer, CAL, SVF- or ADSC-only), we will perform individual subgroup meta-analyses to statistically evaluate how these techniques compare to one another.

We will explore all sources of potential heterogeneity related to study design, participants, interventions, comparators and outcomes. Statistical heterogeneity will be assessed using the chi-square test and quantified with the *I*^2^ statistic. The thresholds for interpretation of the *I*^2^ value will be in accordance with those presented in the Cochrane Handbook for Systematic Reviews of Interventions [16]. An evaluation of between study heterogeneity will inform our decision as to whether a fixed or random effects model is more appropriate for the dataset. If sufficient studies are included in the meta-analysis, we will interrogate the accuracy of our overall outcome estimate using a sensitivity analysis [20].

Continuous outcomes will be analysed using either mean differences or standardised mean differences (with 95% confidence intervals (CIs)); dichotomous data will be analysed using risk ratios (with 95% CIs); time-to-event data will be analysed using hazard ratios (with 95% CIs).

In the event that a meta-analysis is not appropriate, we will combine the results of all included studies in a qualitative synthesis with reference to our primary and secondary outcomes. In this narrative evaluation, we will comment on whether the efficacy and safety of AFG appears to vary according to the intervention subgroups defined above.

## Discussion

In light of the growing popularity of autologous fat grafting for wound healing, a systematic appraisal of the available evidence is timely. Although preclinical studies in both animal and in vitro models are encouraging, whether AFG improves wound healing outcomes in the clinical setting has yet to be conclusively established.

Should AFG be observed to improve wound healing, we hope to identify which technique is superior and whether this varies according to patient- and wound-specific factors. Similarly, we hope to draw meaningful comparisons between AFG interventions and alternative wound management options in an attempt to demonstrate which approach is more effective. Together, these findings should help inform clinical practice when selecting the optimal treatment modality on a case-by-case basis.

We will qualify the strength of our conclusions using a robust and validated methodology for appraising the quality of evidence for each summary outcome measure. We will discuss all relevant methodological issues and areas of uncertainty within the current literature with the intention that these observations are able to guide future research.

## Appendix

**Table 2:** Example free-text term stepwise search strategy

Search 1	(fat graft* OR fat transf* OR fat inject*) AND (wound heal* OR wound management OR wound treat*)
Search 2	(adipose graft* OR adipose stem cell* OR adipose derived stem cell* OR ASC* OR ADSC*) AND (wound heal* OR wound management OR wound treat*)
Search 3	(lipofill* OR lipotransf* OR lipomodell*) AND (wound heal* OR wound management OR wound treat*)
Search 4	(fat graft* OR fat transf* OR fat inject*) AND (ulcer heal* OR ulcer management OR ulcer treat*)
Search 5	(adipose graft* OR adipose stem cell* OR adipose derived stem cell* OR ASC* OR ADSC*) AND (ulcer heal* OR ulcer management OR ulcer treat*)
Search 6	(lipofill* OR lipotransf* OR lipomodell*) AND (ulcer heal* OR ulcer management OR ulcer treat*)

## Abbreviations

ADSC: Adipose derived stem cell
AFG: Autologous fat grafting
CAL: Cell-assisted lipotransfer
CI: Confidence interval
CONSORT: Consolidated Standards of Reporting Trials
GRADE: Grading of Recommendations, Assessment, Development and Evaluation
MeSH: Medical Subject Headings
MSC: Mesenchymal stem cell
MOOSE: Meta-Analysis of Observational Studies in Epidemiology
PRISMA: Preferred Reporting Items for Systematic Reviews and Meta-Analyses
PRP: Platelet-rich plasma
RCT: Randomised control trial
SVF: Stromal vascular fraction
